# Draft Genome Sequence of *Zymomonas mobilis* ZM481 (ATCC 31823)

**DOI:** 10.1128/genomeA.00193-16

**Published:** 2016-04-07

**Authors:** Ning Zhao, Yongxu Pan, Hongguang Liu, Zhen Cheng

**Affiliations:** a Institute of Molecular Medicine, College of Life and Health Sciences, Northeastern University, Shenyang, China; b Molecular Imaging Program at Stanford, Stanford University, Palo Alto, California, USA

## Abstract

*Zymomonas mobilis* ZM481 (ATCC 31823) is an ethanol-tolerant strain that can produce the highest level of ethanol in *Z. mobilis* from glucose in the shortest time. Here, we report a draft genome sequence of ZM481, which can help us understand the genes related to the ethanol tolerance of this strain.

## GENOME ANNOUNCEMENT

*Zymomonas mobilis* quickly metabolizes glucose to ethanol via the unique Entner-Doudoroff pathway, which improves some of the most important technoeconomic factors of production, ethanol yield, and productivity, because of low biomass accumulation and high specific surface area for sugar uptake during fermentation (1). However, as one of the most important bottlenecks, poor tolerance to the high level of accumulated ethanol during fermentation makes it hard to use *Z. mobilis* in industrial production. *Z. mobilis* ATCC 31823 (ZM481) is a high-yielding mutant strain derived from *Z. mobilis* ATCC 31821 (ZM4), which cannot only produce the highest level of ethanol in the shortest time but also get better ethanol tolerance than the original strain (2). These advantages warrant ZM481 as a potential strain for large-scale production of ethanol under high-gravity fermentation. Here, we aim to study the genome information of ZM481 and the genomic differences between ZM481 and ZM4 to understand the genes related to ethanol tolerance.

We sequenced the genome of ZM481 using the Illumina HiSeq 2500 PE126. A library with a fragment length of 500 bp was constructed, and a total of 1 Gbp of clean data from paired-end reads were generated. The genome was assembled using SOAP*denovo* 2.04 (3), and 500× genome coverage resulted in a final assembly of 2,200,722 bp, with a G+C content of 46.06%. The draft genome consists of 30 scaffolds of >500 bp, and the *N_50_* is 385,689 bp. Tandem repetitive sequences were analyzed using Tandem Repeats Finder 4.04 (4), and 56 tandem repeats were found in the genome. Protein-coding sequences were predicted by Glimmer 3.02 (5), and 86.3% of the genome was composed of coding genes. Next, we used BLAST (http://blast.ncbi.nlm.nih.gov/) to accomplish functional annotation by comparing coding gene with nonredundant protein sequences from KEGG (6), Swiss-Prot (7), COG (8), IPS (9), and CAZy (10). rRNA, tRNA, and small RNA (sRNA) genes were detected by RNAmmer 1.2 (11), tRNAscan-SE 1.23 (12), and Rfam 12.0 (13), respectively. The entire genome of ZM481 contains 5 rRNA, 44 tRNA, and 2 sRNA genes.

Because ZM481 was derived from chemical mutagenesis of ZM4, whose genome was completely sequenced and well annotated in 2005 (14), the genomic differences between ZM481 and ZM4 were investigated. Compared with ZM4, single-nucleotide polymorphisms (SNPs) and insertion-deletions (indels) of ZM481 were analyzed via MUMmer 3.22 (15) and LASTZ 1.02.00 (16). The results showed that 125 SNPs (85.62%) localized in the coding regions, and 21 SNPs (14.38%) fell into noncoding regions. No indels were found in the whole genome of ZM481. Synteny analysis of the ZM481 and ZM4 genomes indicated that they were almost the same. These results highlight that SNPs take major responsibility for the ethanol tolerance of ZM481.

### Nucleotide sequence accession numbers.

This whole-genome shotgun project has been deposited at DDBJ/ENA/GenBank under the accession no. LSFP00000000. The version described in this paper is the first version, LSFP01000000.
